# Targeting the IspD Enzyme in the MEP Pathway: Identification of a Novel Fragment Class

**DOI:** 10.1002/cmdc.202100679

**Published:** 2022-01-11

**Authors:** Eleonora Diamanti, Mostafa M. Hamed, Antoine Lacour, Patricia Bravo, Boris Illarionov, Markus Fischer, Matthias Rottmann, Matthias Witschel, Anna K. H. Hirsch

**Affiliations:** ^1^ Helmholtz Institute for Pharmaceutical Research (HIPS) Helmholtz Centre for Infection Research (HZI) Saarland University Campus E8.1 66123 Saarbrücken Germany; ^2^ Helmholtz International Lab for Anti-Infectives Saarland University Campus E8.1 66123 Saarbrücken Germany; ^3^ Department of Pharmacy Saarland University Campus E8.1 66123 Saarbrücken Germany; ^4^ Swiss Tropical and Public Health Institute Socinstrasse 57 4002 Basel Switzerland; ^5^ Universität Basel Petersplatz 1 4003 Basel Switzerland; ^6^ Hamburg School of Food Science University of Hamburg Grindelallee 117 20146 Hamburg Germany; ^7^ BASF-SE Carl-Bosch-Strasse 38 67056 Ludwigshafen Germany

**Keywords:** MEP pathway, IspD, fragment, *Plasmodium falciparum*, drug discovery

## Abstract

The enzymes of the 2‐*C*‐methylerythritol‐d‐erythritol 4‐phosphate (MEP) pathway (MEP pathway or non‐mevalonate pathway) are responsible for the synthesis of universal precursors of the large and structurally diverse family of isoprenoids. This pathway is absent in humans, but present in many pathogenic organisms and plants, making it an attractive source of drug targets. Here, we present a high‐throughput screening approach that led to the discovery of a novel fragment hit active against the third enzyme of the MEP pathway, *Pf*IspD. A systematic SAR investigation afforded a novel chemical structure with a balanced activity–stability profile (**16**). Using a homology model of *Pf*IspD, we proposed a putative binding mode for our newly identified inhibitors that sets the stage for structure‐guided optimization.

## Graphical abstract

**Screening of a part** of the BASF library against the *Pf*IspD enzyme led to the identification of a pyrrole as an underexplored chemical scaffold that is able to target the third enzyme in the MEP pathway. A detailed structure–activity relationship (SAR) study around the fragment hit compound helped us to successfully improve the potency as well as the physicochemical properties.

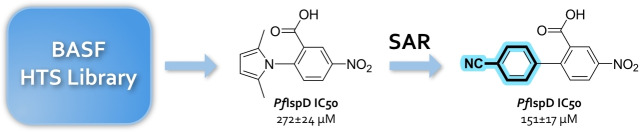

The 2‐*C*‐methylerythritol‐d‐erythritol 4‐phosphate (MEP) pathway, consists of seven enzymes, and is an essential biosynthetic pathway for the production of isopentenyl diphosphate (IDP) and its isomer dimethylallyl diphosphate (DMADP) both of which are universal building blocks of isoprenoids, a large and structurally diverse group of natural products with crucial physiological functions.^[1]^ As the MEP pathway is absent in humans, but essential in most Gram‐negative bacteria, *Mycobacterium tuberculosis* and *Plasmodium falciparum*, the parasite responsible for malaria, it is an attractive source of anti‐infective drug targets.^[2]^ Inhibitors able to target this pathway have the advantage to exhibit a novel mechanism of action without target‐based side effects. Nevertheless, despite the important functions served by the MEP pathway few inhibitors have been reported so far.^[3]^ Importantly, fosmidomycin,^[4]^ a potent inhibitor of the second enzyme of the MEP pathway, IspC or DXR, has undergone phase II clinical trials as antimalarial chemotherapeutic agent in combination with clindamycin and piperaquine, validating the enzymes of the MEP pathway as drug targets.^[5]^ In the present study, we focused our attention on IspD, alternatively known as MEP cytidyltransferase or ygbP protein, that is the third enzyme in the MEP pathway.^[6]^ IspD catalyzes the formation of 4‐diphosphocytidyl‐2‐*C*‐methylerythritol (CDP‐ME) from MEP and cytidine triphosphate CTP in the presence of Mg^2+^, with the release of inorganic diphosphate (PP_i_) (Figure 1).^[7]^

**Figure 1:**
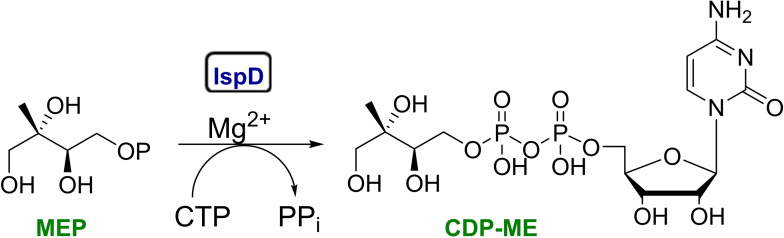
Reaction catalyzed by the IspD protein. MEP: 2‐*C*‐methylerythritol‐d‐erythritol 4‐phosphate; CDP‐ME: 4‐diphosphocytidyl‐2‐*C*‐methylerythritol.

Looking at the IspD inhibitors, to date only a few compounds have been reported.^[8]^ Aiming to enlarge the portfolio of IspD inhibitors and particularly of *Plasmodium* IspD (*Pf*IspD), we performed a high‐throughput screening (HTS), using the proprietary BASF library of about 100,000 diverse selected compounds. The search for novel antimalarial compounds endowed with a novel mechanism of action has a continuous and long history of research. Although novel approved treatments and preventions helped to save many lives, malaria is still responsible for more than 400,000 deaths worldwide, mostly young children.^[9]^

Herein we describe the identification of a fragment‐like compound (**16**) able to inhibit *Pf*IspD *in vitro* in the micromolar range and with a suitable physicochemical profile (Figure 2).

**Figure 2:**
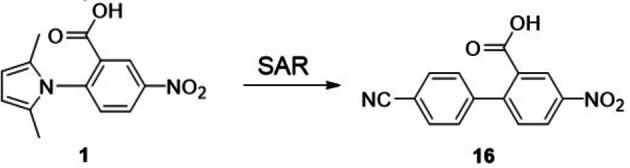
Chemical structures of compounds reported in the present study.

The screening performed against *Pf*IspD led to the identification of compound **1**. Despite its moderate activity it attracted our attention due to its fragment‐like size and modular structure that lends itself to chemical modification. In order to identify structural features that are critical for *Pf*IspD inhibition, we followed a classical structure–activity relationship (SAR) study, as we could not rely on any structural information about the protein. High‐resolution structures are only available for *Escherichia coli* IspD,^[10]^ besides additional structures from non‐pathogenic organisms.^[11]^ We therefore, conducted a focused SAR study with two inter‐related objectives: *i*) find a replacer for the pyrrole ring as it is known to be a structural alert,^[12]^ *ii*) validate the fragment hit by improving the potency and the physicochemical profile for further optimization. With these goals in mind, we initially started our exploration by keeping the core molecule constant and varying only the terminal nitro group (**1**–**8**); while, the second subset of molecules (**9**–**18**) includes modifications around the pyrrole ring.

Half‐maximal inhibitory concentration (IC_50_) values against purified *Pf*IspD are reported in Table 1, while Table 2 summarizes the second set. Details about the assay are reported in the Supporting Information, section 3.

**Table 1:** Inhibition values by 5‐substituted 2‐pyrrol‐1‐benzoic acid derivatives (**1**–**8**) determined using the coupled photometric assay with purified *Pf*IspD.

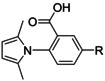		
Compd	R	IC_50_ [μm]*
**1**	NO_2_	271±24
**2**	H	>500
**3**	Cl	263±35
**4**	Br	117±20
**5**	I	208±37
**6**	CH_3_	>500
**7**	−OCH_3_	>500
**8**	−NHCOCH_3_	>500

**Table 2:** [*] IC_50_ values were obtained from two independent experiments.Table 2 Inhibition values by 2‐substituted‐5‐nitrobenzoic acid derivatives (**9**–**18**) determined with the coupled photometric assay using purified *Pf*IspD.

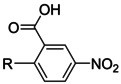		
Compd	R	IC_50_ [μm]*
**9**	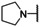	264±30
**10**	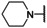	225±27
**11**	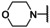	>500
**12**	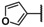	>500
**13**	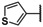	>500
**14**	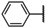	>500
**15**	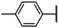	277±56
**16**	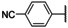	151±17
**17**	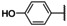	>500
**18**	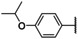	280±55

Compounds **1**–**8** were synthesized following the classical Paal–Knorr pyrrole condensation by refluxing 5‐substitued anthranilic acid in toluene with 1.5 eq of 2,5‐hexanedione in the presence of molecular sieves (Scheme S1).^[13]^

Based on our previously reported discovery of azolopyrimidines^[8a]^ and pseudilins^[14]^ as halogenated and allosteric modulators of the enzyme IspD, we were intrigued to also evaluate the influence of halogens in our new scaffold, leading to derivatives **3**–**5**. In fact, while for the azolopyrimidine scaffold only activity against the plant *Arabidopsis thaliana* (*At*IspD) is reported, the pseudilin‐type inhibitors showed potency against the malaria parasite too, *in vitro* and in cell‐based assays. Specifically, in our series of compounds, the best halogen turned out to be bromine (**4**) that is slightly more potent than hit **1** and about two‐fold more potent than its iodo derivative **5**. By contrast, activity is completely lost upon introduction of an electron‐donating group (**6**–**8**) and also a short elongation with an acetamide group **8** is not tolerated. The drop in activity observed for compound **2** suggests the influence played by a substituent in position 5 of the phenyl ring within this subset of derivatives.

Next, we moved our attention to the pyrrole core. For consistency, we maintained the initial 5‐nitrobenzoic acid scaffold, having the 2‐position occupied by a small but diverse library of aliphatic and substituted aromatic rings (Table 2). For the synthesis of this series of compounds, we relied on the classical Suzuki cross‐coupling reaction between 2‐bromo‐5‐nitrobenzoic acid and the respective boronic acid derivatives (Scheme S2).

Interestingly, the pyrrolidine **9** and the piperidine **10** could replace the dimethyl pyrrole ring without a significant loss in activity. Introducing another heteroatom in the piperidine ring to give the morpholine **11** led to a further decrease in activity. Other 5‐membered heterocyclic rings such as the furan **12** and the thiophene **13** were not beneficial for the activity. Although, the unsubstituted phenyl **14** did not show significant activity, further substitution on the ring seemed to be beneficial. Substituents of variable nature such as the methyl **15** and the nitrile **16** restored the activity of the unsubstituted phenyl, suggesting there is room for further modification on this side of the molecule. The nitrile derivative **16** showed almost two‐fold higher potency than its methyl analogue **15**. Exploring other polar groups such as the hydroxy **17** was not useful, hinting that the electronic effects exerted by the substituents may affect the activity. Remarkably, further growing on the hydroxyl with an isopropyl **18** regained the activity, indicating some space that could be available to modulate the activity.

To gain further insights into this class of compounds, we built a homology model for *Pf*IspD using *Ec*IspD (PDB 1I52) as a template and docked our compounds into the substrate binding pocket (Figure 3). The docked pose of hit compound **1** (Figure 3a) shows that the carboxylic group forms two H bonds with Lys207 and Ile205 in the binding pocket. Both H bonds were also seen in the case of **4** and **16** with a substituted phenyl instead of the dimethyl pyrrole. Another H bond is formed between the compounds having the terminal NO_2_ group and Arg429.

**Figure 3:**
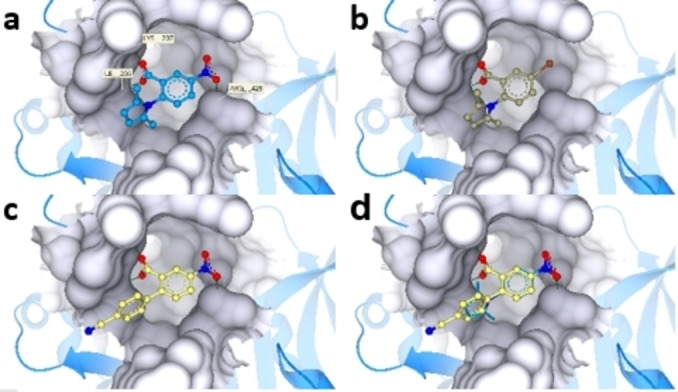
Docked poses of compounds **1**, **4**, and **16** in the homology model of *Pf*IspD using SeeSAR 11.0. **a**) The carboxylic group of **1** (blue) forms two H bonds (green dotted lines) with Lys207 and Ile205 and the NO_2_ group is engaged in a H bonding interaction with Arg429. **b**) The carboxylic group in **4** (beige) forms the same H bonds as for compound **1. c**) Compound **16** (yellow) forms the same H bonds with Lys207, Ile205 and Arg429 as the hit compound **1. d)** Overlay of the docked poses of compounds **1** and **16**.

Finally, as the affinity for the target is not the only aspect to be considered during fragment‐based drug discovery, we also focused our attention on the physicochemical properties of our compounds (see Table 3).^[15]^ Despite compound **4** showing the best potency in our series of compounds, we do not consider it a suitable candidate for further fragment growing as the lipophilic ligand‐efficiency (LLE) parameter is not ideal. Most probably, the better IC_50_ value is due to the higher cLogP value. Conversely, with compound **16** we have a good balance in all the ligand efficiency scores evaluated. Of note, having an aromatic core with a nitrile substituent as in **16**, has several advantages compared to the pyrrole liability.^[16]^

**Table 3:** [*] IC_50_ values were obtained from two independent experiments.Table 3 Summary of ligand‐efficiency scores calculated on StarDrop version: 7.0.1.29911.

	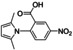	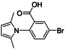	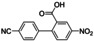
Compd	**1**	**4**	**16**
*Pf*IspD IC_50_ [μm]	271±24	117±20	151±17
cLogP	1.79	3.84	1.75
MW^[a]^	262.3	294.1	270.2
HA^[b]^	19	17	20
LE^[c]^	0.26	0.32	0.26
LLE^[d]^	1.77	0.085	2.072

[a] Molecular weight. [b] Non‐hydrogen atom. [c] Ligand efficiency. [d] Lipophilic ligand efficiency.

In conclusion, the present report describes the identification of compound **16** as an optimized fragment hit, targeting *Pf*IspD with high potential for further fragment growing and optimization.

## Conflict of interest

The authors declare no conflict of interest.

## Supporting information

As a service to our authors and readers, this journal provides supporting information supplied by the authors. Such materials are peer reviewed and may be re‐organized for online delivery, but are not copy‐edited or typeset. Technical support issues arising from supporting information (other than missing files) should be addressed to the authors.

Supporting Information

## Data Availability

The data that support the findings of this study are available in the supplementary material of this article.
