# Supplementary material for: Targeting the IspD Enzyme in the MEP Pathway: Identification of a Novel Fragment Class
Source: ChemMedChem. 2022 Jan 11;17(5):e202100679. doi: 10.1002/cmdc.202100679 (PMC9305118; doi:10.1002/cmdc.202100679)
Supplement: Supplementary file 1 — Supporting Information [file CMDC-17-0-s001.pdf]

ChemMedChem

Supporting Information

Targeting the IspD Enzyme in the MEP Pathway: Identification of a Novel Fragment Class

Eleonora Diamanti[+], Mostafa M. Hamed[+], Antoine Lacour, Patricia Bravo, Boris Illarionov, Markus Fischer, Matthias Rottmann, Matthias Witschel, and Anna K. H. Hirsch*

Table of Contents

1 LIST OF ABBREVIATIONS 3

2 EXPERIMENTAL 3

2.1 CHEMISTRY 3

2.1.1 Materials and Methods 3

2.1.2 Preparative RP-HPLC purifications 3

2.1.3 Synthetic Schemes 4

2.1.4 Experimental procedures 5

3 Biochemistry 12

3.1 *Pf*IspD assay 12

4 Molecular modeling 13

5 ^{1}H- AND ^{13}C-NMR SPECTRA OF SOME REPRESENTATIVE COMPOUNDS 13

6 REFERENCES 21

Internal

1 LIST OF ABBREVIATIONS

Acetonitrile (CH_3CN), formic acid (HCOOH), dimethylsulfoxide (DMSO), ethyl acetate (EtOAc), sodium carbonate (Na_2CO_3), water (H_2O), magnesium sulfate ($MgSO_4$), hour (h), minutes (min), ON (overnight).

2 EXPERIMENTAL

2.1 CHEMISTRY

2.1.1 Materials and Methods

Starting materials and solvents were purchased from commercial suppliers, and used without further purification. All chemical yields refer to purified compounds and were not optimized. Reaction progress was monitored using TLC silica gel 60 F_{254} aluminum sheets, and visualization was accomplished by UV at 254 nm. Column chromatography was performed using the automated flash chromatography system CombiFlash® Rf (Teledyne Isco) equipped with RediSepRf silica columns. Preparative RP-HPLC was perofmed using an UltiMate 3000 Semi-Preparative System (Thermo Fisher Scientific) with nucleodur® C18 Gravity (250 mm × 16 mm, 5 µm) column. ^{1}H and ^{13}C NMR spectra were recorded as indicated on a Bruker Avance Neo 500 MHz (^{1}H, 500 MHz; ^{13}C, 126 MHz) with prodigy cryoprobe system. Chemical shifts were recorded as δ values in ppm units and referenced against the residual solvent peak (DMSO-d_6, δ = 2.50, 39.52, acetone-d_6: δ = 2.05, 29.84). Splitting patterns describe apparent multiplicities and are designated as s (singlet), br s (broad singlet), d (doublet), dd (doublet of doublet), t (triplet), q (quartet), m (multiplet). Coupling constants (*J*) are given in hertz (Hz). Low resolution mass analytics and purity control of final compounds was carried out using an Ultimate 3000-MSQ LCMS system (Thermo Fisher Scientific) consisting of a pump, an autosampler, MWD detector and a ESI quadrupole mass spectrometer. Purity of all compounds used in biochemical assays was ≥ 95%. High resolution mass spectra were recorded on a ThermoFisher Scientific (TF, Dreieich, Germany) Q Exactive Focus system equipped with heated electrospray ionization (HESI)-II source. Final products were dried at high vacuum.

2.1.2 Preparative RP-HPLC purifications

Purifications via preparative RP-HPLC were carried out using the following condition:

Internal

Gradient 5–100% CH_3CN + 0.05% HCOOH in water + 0.05% HCOOH in 53 min at a flow rate of 10 mL/min.

The sample was dissolved in DMSO and manually injected to the HPLC system.

2.1.3 **Synthetic Schemes**

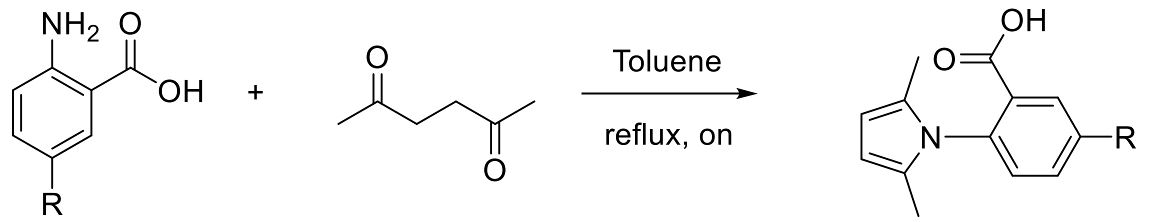

1a, R = NO_2
2a, R = H
3a, R = Cl
4a, R = Br
5a, R = I
6a, R = CH_3
7a, R = OCH_3

1, R = NO_2 (11%)
2, R = H (26%)
3, R = Cl (15%)
4, R = Br (46%)
5, R = I (48%)
6, R = CH_3 (44%)
7, R = OCH_3 (63%)

Scheme S1: General procedure for the synthesis of compounds **1**—**7**.

Internal

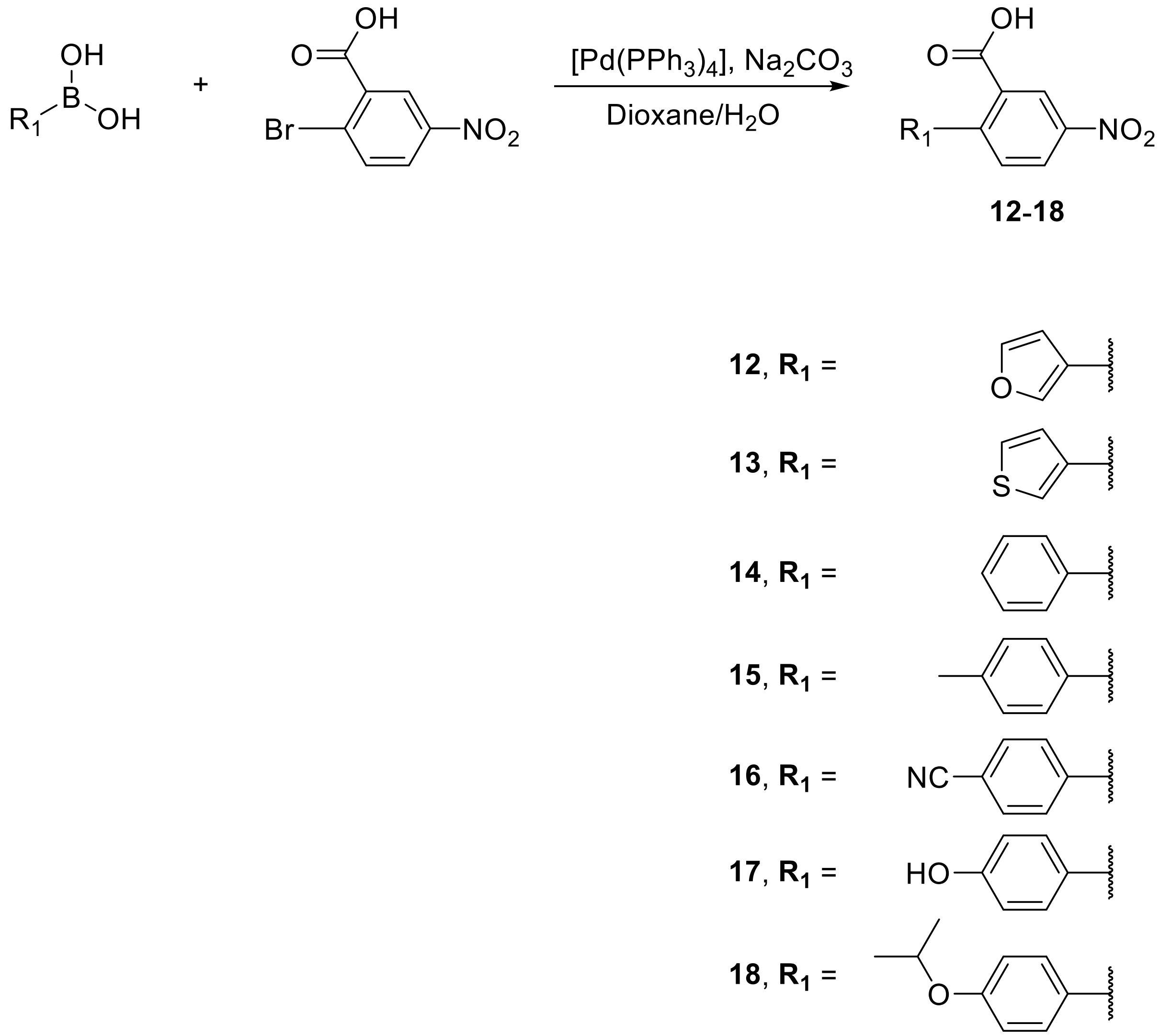

Scheme S2: General procedure for the synthesis of compounds **12**–**18**.

2.1.4 **Experimental procedures**

Compounds **8**, **9**, **10** and **11** were commercially available and were purchased directly. Compound **8** (CAS: 332898-63-2) was purchased from abcr. Compounds **9** (CAS: 19555-48-7), **10** (CAS: 42106-50-3) and **11** (CAS: 4036-83-3) were purchased from Enamine.

General Procedure A (GPA): Paal-Knorr condensation.

To a stirred solution of 5–substituted 2-amino benzoic acid in toluene (0.1 M), 1.2 eq of 2,5–hexanedione were added. The reaction mixture was stirred under reflux overnight in the presence

Internal

of molecular sieves. Once cooled, the solvent was evaporated, and the crude mixture was purified via preparative HPLC.

General Procedure B (GPB): **Suzuki coupling**.

In a microwave vial containing compound 2-bromo-5-nitrobenzoic acid (1 mmol), boronic acid derivative (1.3 mmol) and [Pd(PPh_3)$_4$] (0.05 mmol), was added the dioxane:H_2O mixture (3 mL, 4:1) and Na_2CO_3 (4 mmol). The reaction was heated in a microwave at 120° C for 1 h. H_2O (30 mL) was added followed by extraction with ethyl acetate (2 x 30 mL). The combined organic layers were dried over $MgSO_4$, filtered and concentrated under reduced pressure. Purification was done using preparative HPLC to yield compounds (**12**–**18**).

2-(2,5-Dimethyl-1*H*-pyrrol-1-yl)-5-nitrobenzoic acid (1)

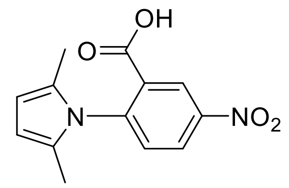

According to GPA, using 2-amino-5-nitrobenzoic acid (0.100 g, 0.55 mmol) and 2,5-hexanedione (0.077 mL, 0.66 mmol) in toluene (5.5 mL) to give, after purification by preparative HPLC (eluted at 40% CH_3CN), the product **1** as slightly red powder (0,015 g, 11%). **^{1}H NMR (500 MHz, DMSO-*d₆*)** *δ* 8.59 (d, *J* = 2.5, 1H), 8.47 (dd, *J* = 8.6, 2.6, 1H), 7.60 (d, *J* = 8.6, 1H), 5.80 (s, 2H), 1.88 (s, 6H). **^{13}C NMR (126 MHz, DMSO-*d₆*)** *δ* 165.5, 146.8, 142.7, 133.4, 132.3, 128.1, 126.9, 125.1, 106.5, 12.5. **HRMS (ESI)** *m/z* calcd for $C_{13}H_{11}N_2O_4$ (*M*-H)$^-$: 259.07243, found: 259.07233.

2-(2,5-Dimethyl-1*H*-pyrrol-1-yl)benzoic acid (2)

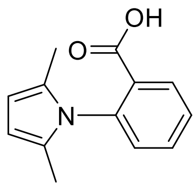

According to GPA, using 2-aminobenzoic acid (0.100 g, 0.73 mmol) and 2,5-hexanedione (0.103 mL, 0.87 mmol) in toluene (7.3 mL) to give, after purification by preparative HPLC (eluted at 55% CH_3CN) the product **2** as red powder (0.040 g, 26%). **^{1}H NMR (500 MHz, DMSO-*d₆*)** *δ* 7.84 (dd, *J* = 7.7, 1.5, 1H), 7.66 (td, *J* = 7.7, 1.6, 1H), 7.56 (td, *J* = 7.6, 1.1, 1H), 5.72 (s, 2H), 5.72 (s, 2H), 1.85 (s, 6H). **^{13}C NMR (126**

Internal

MHz, DMSO-*d*$_6$) *δ* 167.2, 137.05, 132.4, 132.1, 130.3, 129.9, 128.5, 127.8, 105.4, 12.5. **HRMS (ESI)** *m/z* calcd for $C_{13}H_{12}NO_2$ (*M*-H)$^-$: 214.08735, found: 214.08678.

5-Chloro-2-(2,5-dimethyl-1*H*-pyrrol-1-yl)benzoic acid (3)

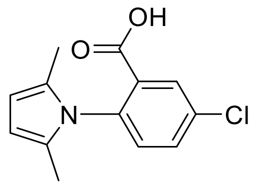

According to GPA, using 2-amino-5-chlorobenzoic acid (0.100 g, 0.58 mmol) and 2,5-hexanedione (0.08 mL, 0. 7 mmol) in toluene (5.8 mL) to give, after purification by preparative HPLC (eluted at 55% CH_3CN) the product **3** as red powder (0,022g, 15%). **^{1}H NMR (500 MHz, DMSO-*d*$_6$)** *δ* 7.85 (d, *J* = 2.4, 1H), 7.72 (dd, *J* = 8.4, 2.5, 1H), 7.31 (d, *J* = 8.4, 1H), 5.73 (s, 2H), 1.85 (s, 6H). **^{13}C NMR (126 MHz, DMSO-*d*$_6$)** *δ* 165.9, 135.8, 134.2, 132.9, 132.3, 131.9, 129.5, 128.0, 105.7, 12.5; **HRMS (ESI)** *m/z* calcd for $C_{13}H_{11}ClNO_2$ (*M*-H)$^-$: 248.04838 found: 248.04796.

5-Bromo-2-(2,5-dimethyl-1*H*-pyrrol-1-yl)benzoic acid (4)

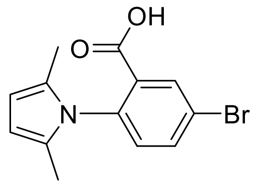

According to GPA, using 2-amino-5-bromobenzoic acid (0.100 g, 0.46 mmol) and 2,5-hexanedione (0.065 mL, 0.55 mmol) in toluene (4.6 mL) to give, after purification by preparative HPLC (eluted at 74% CH_3CN) the product **4** as red oil (0.060 g, 46%). **^{1}H NMR (500 MHz, DMSO-*d*$_6$)** *δ* 13.18 (s, 1H), 7.98 (d, *J* = 2.4, 1H), 7.86 (dd, *J* = 8.4, 2.4, 1H), 7.24 (d, *J* = 8.4, 1H), 5.73 (s, 2H), 1.85 (s, 6H). **^{13}C NMR (126 MHz, DMSO-*d*$_6$)** *δ* 165.8, 136.3, 134.9, 134.4, 132.5, 132.3, 127.9, 121.3, 105.7, 12.5. **HRMS (ESI)** *m/z* calcd for $C_{13}H_{13}BrNO_2$ (*M*+H)$^+$: 294.01242, found: 294.01175.

5-Iodo-2-(2,5-dimethyl-1*H*-pyrrol-1-yl)benzoic acid (5)

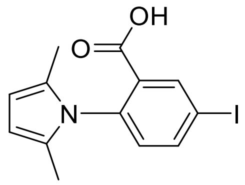

According to GPA, using 2-amino-5-iodobenzoic acid (0.100 g, 0.38 mmol), 2,5-hexanedione (0.053 mL, 0.45 mmol) in Toluene (3.8 mL) to give, after purification by preparative HPLC (eluted at 75% CH_3CN)

Internal

the product **6** as red powder (0.062 g, 48%). **^{1}H NMR (500 MHz, DMSO-*d$_6$*)** *δ* 8.12 (d, *J* = 2.1, 1H), 8.00 (dd, *J* = 8.2, 2.1, 1H), 7.06 (d, *J* = 8.2, 1H), 5.72 (s, 2H), 1.85 (s, 6H). **^{113}C NMR (126 MHz, DMSO-*d$_6$*)** *δ* 166.2, 141.2, 138.6, 137.1, 134.8, 132.8, 128.4, 106.13, 94.8, 12.9. **HRMS (ESI)** *m/z* calcd for $C_{13}H_{13}INO_2$ $(M+H)^+$: 341.99855, found: 341.99792.

2-(2,5-Dimethyl-1*H*-pyrrol-1-yl)-5-methylbenzoic acid (6)

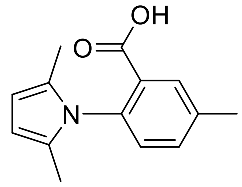

According to GPA, using 2-amino-5-methylbenzoic acid (0.100 g, 0.66 mmol), 2,5-hexanedione (0.09 mL, 0.8 mmol) in Toluene (6.6 mL) to give, after purification by preparative HPLC (eluted at 60% CH_3CN) the product **6** as red powder (0.065 g, 44%). **^{1}H NMR (500 MHz, DMSO-*d$_6$*)** *δ* 7.65 (d, *J* = 1.6, 1H), 7.45 (dd, *J* = 8.0, 1.7, 1H), 7.12 (d, *J* = 7.9, 1H), 5.70 (s, 2H), 2.41 (s, 3H), 1.84 (s, 6H). **^{13}C NMR (126 MHz, DMSO-*d$_6$*)** *δ* 167.3, 138.1, 134.5, 132.6, 131.9, 130.3, 130.0, 127.9, 105.2, 20.5, 12.6. **HRMS (ESI)** *m/z* calcd for $C_{14}H_{16}NO_2$ $(M+H)^+$: 230.11756, found: 230.11723.

2-(2,5-Dimethyl-1*H*-pyrrol-1-yl)-5-methoxybenzoic acid (7)

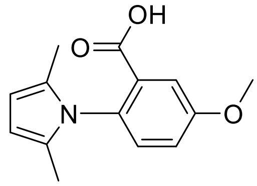

According to GPA, using 2-amino-5-methoxybenzoic acid (0.100 g, 0.59 mmol), 2,5-hexanedione (0.084 mL, 0.71 mmol) in toluene (5.9 mL) to give, after purification by preparative HPLC (eluted at 72% CH_3CN) the product **7** as red powder (0.090 g, 63%). **^{1}H NMR (500 MHz, DMSO-*d$_6$*)** *δ* 7.32 (d, *J* = 2.8, 1H), 7.20 (dd, *J* = 8.6, 2.8, 1H), 7.16 (d, *J* = 8.6, 1H), 5.69 (s, 2H), 3.85 (s, 3H), 1.84 (s, 6H). **^{13}C NMR (126 MHz, DMSO-*d$_6$*)** *δ* 166.9, 158.5, 133.3, 131.5, 129.6, 128.1, 117.5, 114.4, 105.1, 55.6, 12.6. **HRMS (ESI)** *m/z* calcd for $C_{14}H_{14}NO_3$ $(M-H)^-$: 244.09792, found: 244.09744.

5-Acetamido-2-(2,5-dimethyl-1*H*-pyrrol-1-yl)benzoic acid (8)

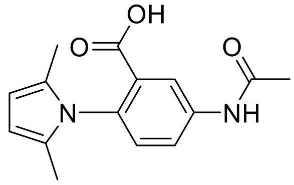

Internal

CAS: 332898-63-2

5-Nitro-2-(pyrrolidin-1-yl)benzoic acid (9)

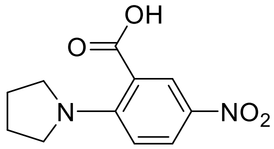

CAS: 19555-48-7

5-Nitro-2-(piperidin-1-yl)benzoic acid (10)

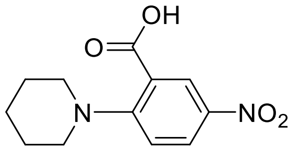

CAS: 42106-50-3

2-Morpholino-5-nitrobenzoic acid (11)

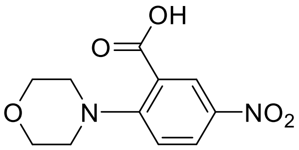

CAS: 4036-83-3

2-(Furan-3-yl)-5-nitrobenzoic acid (12)

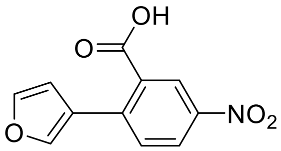

Compound **12** was synthesized according to GPB by reacting 2-bromo-5-nitrobenzoic acid with furan-3-ylboronic acid. The crude product was purified by preparative HPLC (eluted at 45% CH_3CN, pale yellow solid, 59 % yield). **^{1}H NMR (500 MHz, Acetone-*d$_6$*)** δ 8.57 (d, *J* = 2.5, 1H), 8.38 (dd, *J* = 8.6, 2.5, 1H), 7.97 (s, 1H), 7.82 (d, *J* = 8.6, 1H), 7.67 (t, *J* = 1.5, 1H), 6.76 (d, *J* = 1.0, 1H). **^{13}C NMR (126 MHz, Acetone-*d$_6$*)** δ 168.1, 147.3, 144.4, 142.6, 139.4, 133.4, 132.3, 126.3, 125.2, 124.6, 112.0. **HRMS (ESI)** *m/z* calcd for $C_{11}H_6NO_5$ $(M\text{-H})^-$: 232.02515 found 232.02499.

5-Nitro-2-(thiophen-3-yl)benzoic acid (13)

Internal

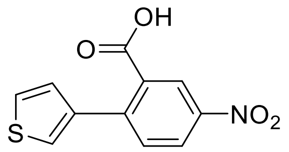

Compound **13** was synthesized according to GPB by reacting 2-bromo-5-nitrobenzoic acid with thiophen-3-ylboronic acid. The crude product was purified by preparative HPLC (eluted at 55% CH_3CN, pale yellow solid, 25 % yield). **^{1}H NMR (500 MHz, Acetone-*d$_6$*)** δ 8.58 (d, *J* = 2.4, 1H), 8.40 (dd, *J* = 8.5, 2.4, 1H), 7.80 (d, *J* = 8.5, 1H), 7.69 (d, *J* = 1.8, 1H), 7.57 (dd, *J* = 4.9, 3.0, 1H), 7.29 (d, *J* = 4.5, 1H). **^{13}C NMR (126 MHz, Acetone-*d$_6$*)** δ 168.1, 147.5, 143.2, 140.2, 133.9, 132.8, 129.1, 126.8, 126.2, 125.5, 125.1. **HRMS (ESI)** *m/z* calcd for $C_{11}H_6NO_4S$ $(M\text{-H})^-$: 248.00230 found 248.00226.

4-Nitro-[1,1'-biphenyl]-2-carboxylic acid (14)

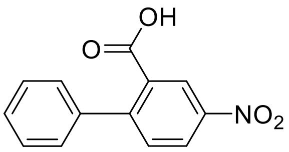

Compound **14** was synthesized according to GPB by reacting 2-bromo-5-nitrobenzoic acid with phenylboronic acid. The crude product was purified by preparative HPLC (eluted at 55% CH_3CN, white solid, 20% yield. **^{1}H NMR (500 MHz, Acetone-*d$_6$*)** δ 8.64 (d, *J* = 2.4, 1H), 8.44 (dd, *J* = 8.5, 2.5, 1H), 7.73 (d, *J* = 8.5, 1H), 7.49 – 7.43 (m, 5H); **^{13}C NMR (126 MHz, Acetone-*d$_6$*)** δ 167.8, 149.1, 147.7, 140.3, 133.9, 133.2, 129.2, 129.2, 126.3, 125.41. **HRMS (ESI)** *m/z* calcd for $C_{13}H_8NO_4$ $(M\text{-H})^-$: 242.04588 found 242.04581.

4'-Methyl-4-nitro-[1,1'-biphenyl]-2-carboxylic acid (15)

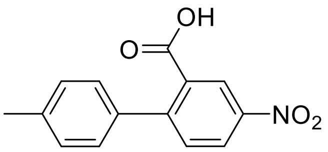

Compound **15** was synthesized according to GPB by reacting 2-bromo-5-nitrobenzoic acid with p-tolylboronic acid. The crude product was purified by preparative HPLC (eluted at 60% CH_3CN, white solid, 58 % yield). **^{1}H NMR (500 MHz, Acetone-*d$_6$*)** δ 8.61 (d, *J* = 2.3, 1H), 8.41 (dd, *J* = 8.5, 2.4, 1H), 7.70 (d, *J* = 8.5, 1H), 7.34 (d, *J* = 8.0, 2H), 7.27 (d, *J* = 7.9, 2H), 2.38 (s, 3H). **^{13}C NMR (126 MHz, Acetone-*d$_6$*)** δ 168.0, 149.0, 147.5, 139.1, 137.3, 133.9, 133.1, 129.8, 129.2, 126.2, 125.3, 21.2. **HRMS (ESI)** *m/z* calcd for $C_{14}H_{10}NO_4$ $(M\text{-H})^-$: 256.06153 found 256.06161.

4'-Cyano-4-nitro-[1,1'-biphenyl]-2-carboxylic acid (16)

Internal

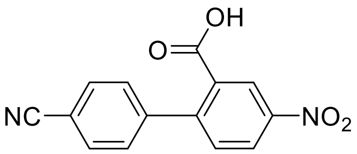

Compound **16** was synthesized according to GPB by reacting 2-bromo-5-nitrobenzoic acid with (4-cyanophenyl)boronic acid. The crude product was purified by preparative HPLC (eluted at 50% CH_3CN, white solid, 25 % yield). **^{1}H NMR (500 MHz, Acetone-*d₆*)** *δ* 8.74 (d, *J* = 2.1, 1H), 8.49 (dd, *J* = 8.4, 2.2, 1H), 7.88 (d, *J* = 8.1, 2H), 7.76 (d, *J* = 8.4, 1H), 7.66 (d, *J* = 8.1, 2H). **^{13}C NMR (126 MHz, Acetone-*d₆*)** *δ* 166.9, 148.4, 147.8, 145.4, 133.4, 133.3, 132.8, 130.3, 126.7, 125.9, 119.2, 112.7. **HRMS (ESI)** *m/z* calcd for $C_{14}H_7N_2O_4$ (*M*-H)$^-$: 267.04113 found 267.04128.

4'-Hydroxy-4-nitro-[1,1'-biphenyl]-2-carboxylic acid (17)

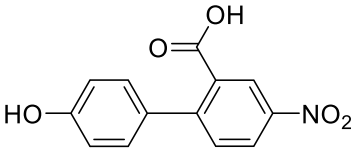

Compound **17** was synthesized according to GPB by reacting 2-bromo-5-nitrobenzoic acid with (4-hydroxyphenyl)boronic acid. The crude product was purified by preparative HPLC (eluted at 45% CH_3CN, beige solid, 62 % yield). **^{1}H NMR (500 MHz, Acetone-*d₆*)** *δ* 8.57 (d, *J* = 2.5, 1H), 8.38 (dd, *J* = 8.5, 2.5, 1H), 7.69 (d, *J* = 8.5, 1H), 7.34 – 7.29 (m, 2H), 6.96 – 6.91 (m, 2H). **^{13}C NMR (126 MHz, Acetone-*d₆*)** *δ* 168.4, 158.9, 148.9, 147.1, 133.7, 132.8, 131.2, 130.7, 126.1, 125.3, 116.2. **HRMS (ESI)** *m/z* calcd for $C_{13}H_8NO_5$ (*M*-H)$^-$: 258.04080 found 258.04082.

4'-Isopropoxy-4-nitro-[1,1'-biphenyl]-2-carboxylic acid (18)

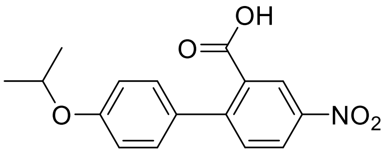

Compound **18** was synthesized according to GPB by reacting 2-bromo-5-nitrobenzoic acid with (4-isopropoxyphenyl)boronic acid. The crude product was purified by preparative HPLC (eluted at 62% CH_3CN, pale yellow solid, 33 % yield). **^{1}H NMR (500 MHz, Acetone-*d₆*)** *δ* 8.59 (s, 1H), 8.40 (d, *J* = 7.6, 1H), 7.71 (d, *J* = 8.3 Hz, 1H), 7.38 (d, *J* = 8.0, 2H), 7.00 (d, *J* = 8.0, 2H), 4.77 – 4.64 (m, 1H), 1.34 (d, *J* = 5.6, 6H). **^{13}C NMR (126 MHz, Acetone-*d₆*)** *δ* 168.2, 159.4, 148.7, 147.2, 133.7, 132.9, 131.9, 130.7, 126.2, 125.3, 116.3, 70.4, 22.3. **HRMS (ESI)** *m/z* calcd for $C_{16}H_{14}NO_5$ (*M*-H)$^-$: 300.08775 found 300.08796.

Internal

3 Biochemistry

3.1 *Pf*IspD assay

Assays were conducted in 384-well plates (Corning) with a transparent flat bottom. Assay buffer A (total volume, 30 µL) contained 200 mM Tris hydrochloride, pH 7.6, 20 mM KCl, 10 mM $MgCl_2$, 10 mM DTT, 1.2 mM NADH, 2 mM ATP, 2 mM phosphoenolpyruvic acid, 0.2 U of pyruvate kinase (PK), 0.2 U of lactate dehydrogenase (LDH), 0.2 U (1.8 µM) IspE from *E. coli* (*Ec*IspE) and 0.004 U (0.050 µM) of IspD protein from *Plasmodium falciparum* (*Pf*IspD). Dilution series were performed with dilution step 1:2 and approximately covered the concentration range of 200 µM to 0.01 µM of tested compounds. The reaction was started by addition of the assay buffer B that contained 200 mM Tris hydrochloride, pH 7.6, 1 mM MEP and 1 mM CTP.

The impact of the tested compounds on auxiliary enzymes in the photometric activity inhibition assay was tested as follows. Test buffer A (total volume, 30 µL) contained 200 mM Tris hydrochloride, pH 7.6, 20 mM KCl, 10 mM $MgCl_2$, 10 mM DTT, 1.2 mM NADH, 2 mM ATP, 2 mM phosphoenolpyruvic acid, 0.2 U of pyruvate kinase (PK), 0.2 U of lactate dehydrogenase (LDH) and 0.004 U (0.02 µM) *Ec*IspE. Dilution series were performed with dilution step 1:2 and approximately covered the concentration range of 200 µM to 0.01 µM of tested compounds. The reaction was started by addition of the assay buffer B that contained 200 mM Tris hydrochloride, pH 7.6, 1 mM CDP-ME.

The reactions were monitored photometrically (room temperature) at 340 nm in a plate reader (SpectraMax5, Molecular Dynamics). Initial rate values were evaluated with a nonlinear regression method using the program Dynafit. [1]

MEP and CDP-ME were used as starting substrate in the IspD and IspE assay, respectively, and were synthesised and purified as described earlier.[2]

All the compounds tested (**1**–**18**) showed no activity against the auxiliary enzyme.

This assay has been used in the initial HTS and as with most enzyme-based approaches, IspD hits may fall under PAIN-subcluster (pan-assay interference compounds), which indicate risk of non-specific interference with the assay, but sometimes also exclude valuable leads. The control reaction via the auxiliary IspE-enzyme done for the most active hits confirm that the described inhibitors are, in fact, selectively inhibiting the target enzyme and not the auxiliary enzymes of the IspD assay. Still, in follow up optimization the potential of non-specific assay interaction should be taken into account for further analogues

Internal

4 Molecular modeling

The *Pf*IspD homology model was constructed using the sequence from 2-*C*-methyl-D-erythritol 4-phosphate cytidylyltransferase from *Plasmodium falciparum* 3D7 (obtained from the NCBI (www.ncbi.nlm.nih.gov, sequence ID: XP_001351000.2, accessed 11/10/2021) and the structure from *E. coli* IspD (accessed 11/10/2021). The model was built using the PHYRE2 online homology modeling program. [3] Previous work showed the 1I52 *E. coli* IspD structure served as a good template.[4] The model created in PHYRE2 using 1I52 as the template structure showed a 99.77% degree of confidence. Compounds of interest were docked into the homology model described above using the docking algorithm provided in SeeSAR 11.0 (BioSolveIT GPBH). Binding site definition was carried out manually from residues selected by SeeSAR's pocket finding function. The binding site selected is analogous to the CDP-ME binding site in the 1I52 *E. coli* IspD structure used as a template.

5 ^{1}H- AND ^{13}C-NMR SPECTRA OF SOME REPRESENTATIVE COMPOUNDS

Compound **12**

Internal

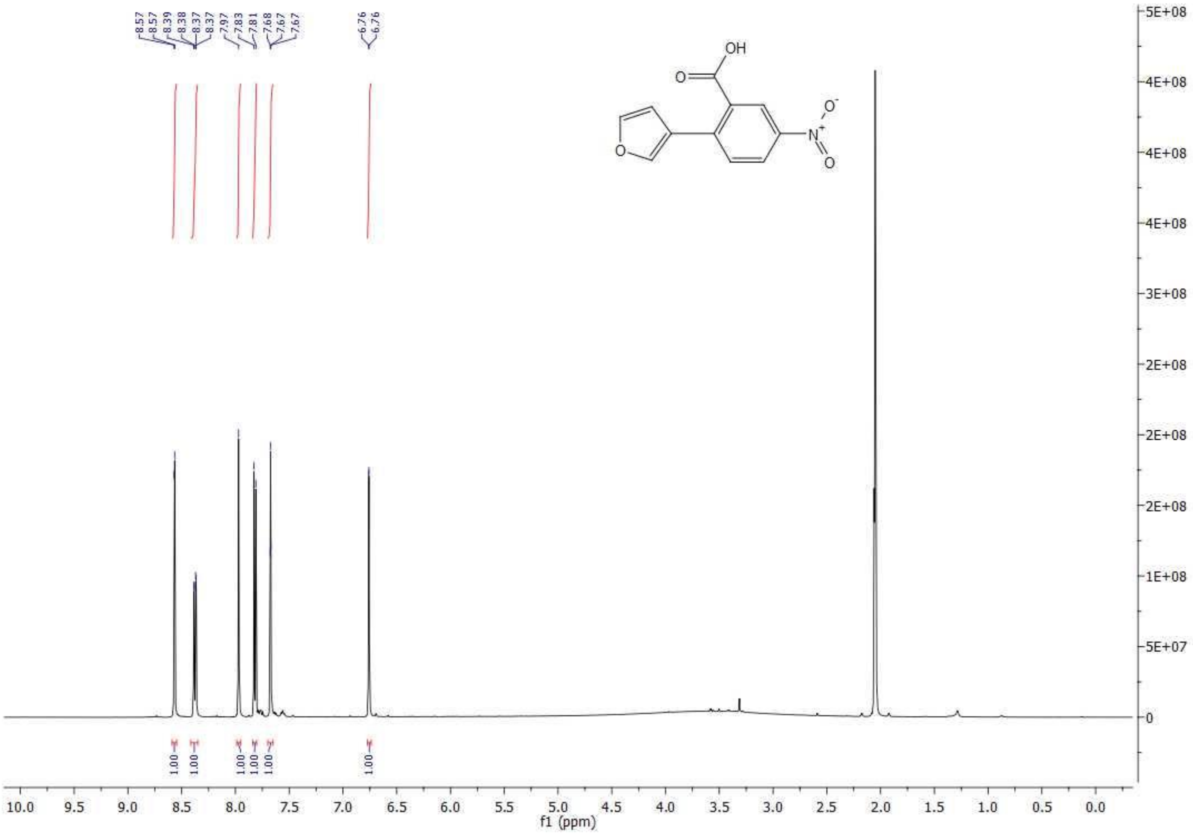

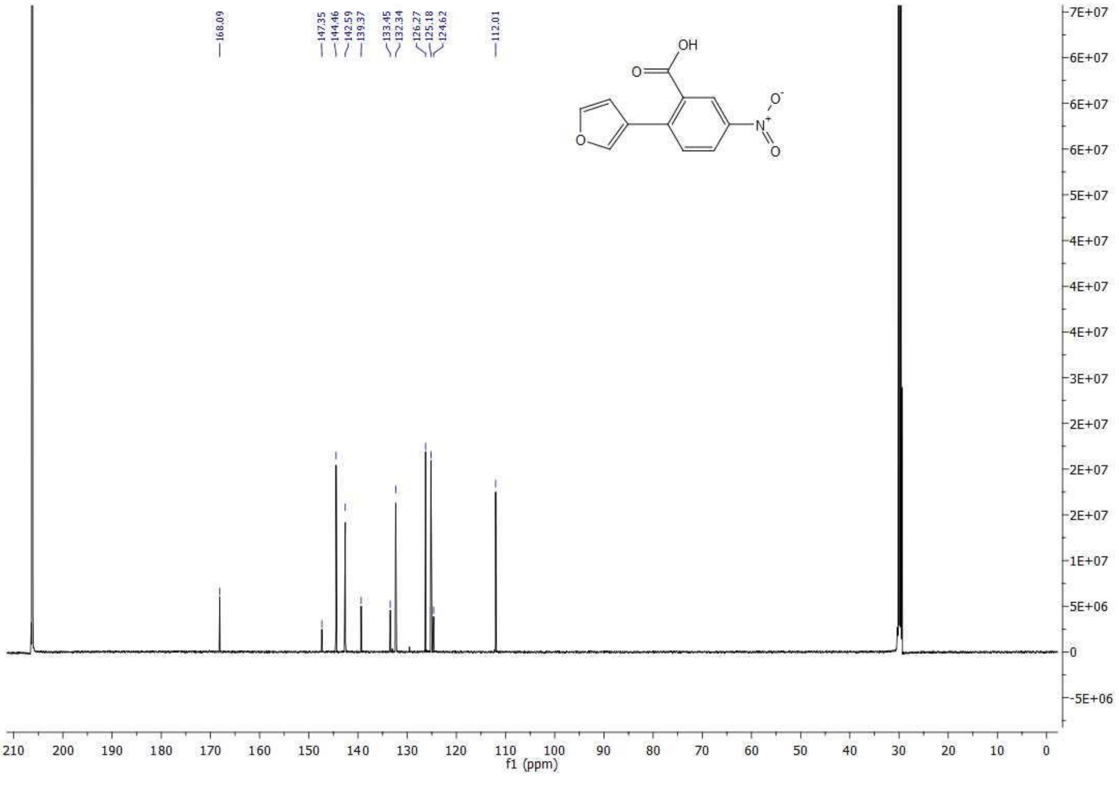

Compound **13**

Internal

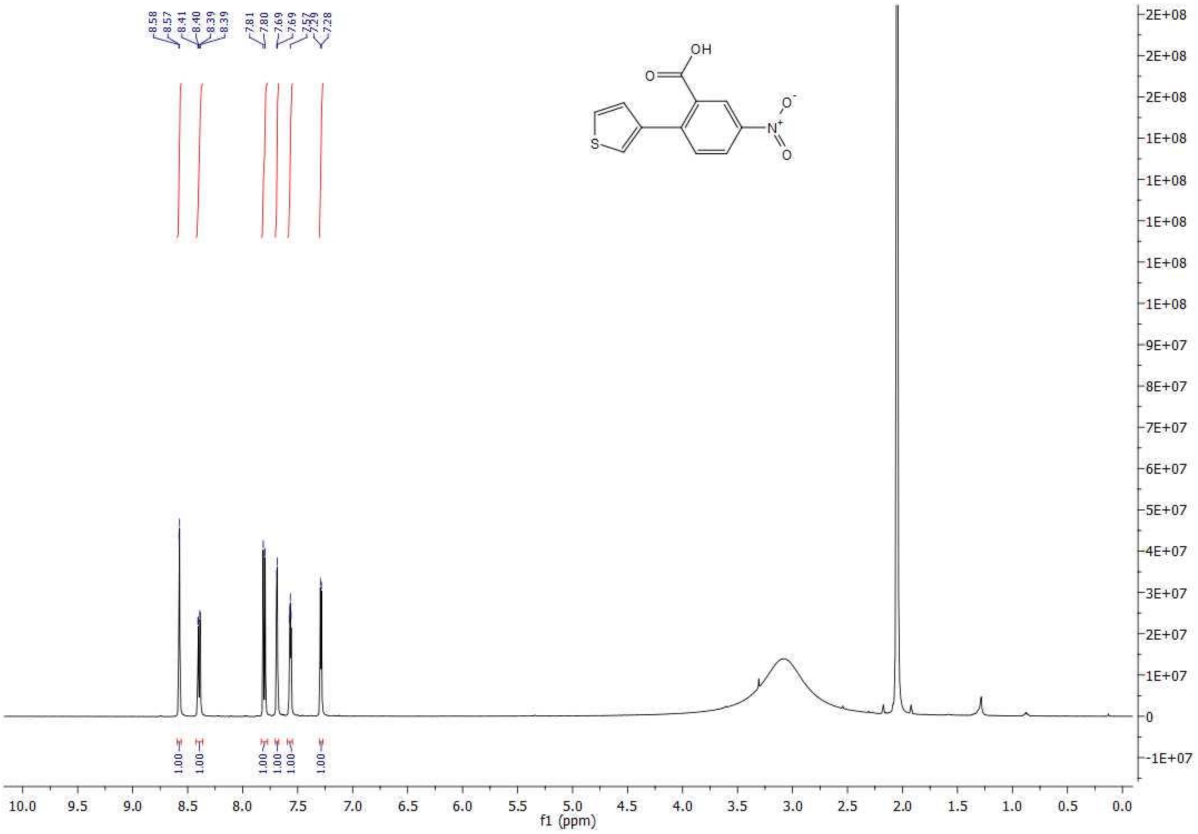
8.58
8.57
8.41
8.40
8.39
8.39
7.81
7.80
7.69
7.69
7.57
7.29
7.28
OH
O
S
N+
O-
1.00
1.00
1.00
1.00
1.00
1.00
f1 (ppm)

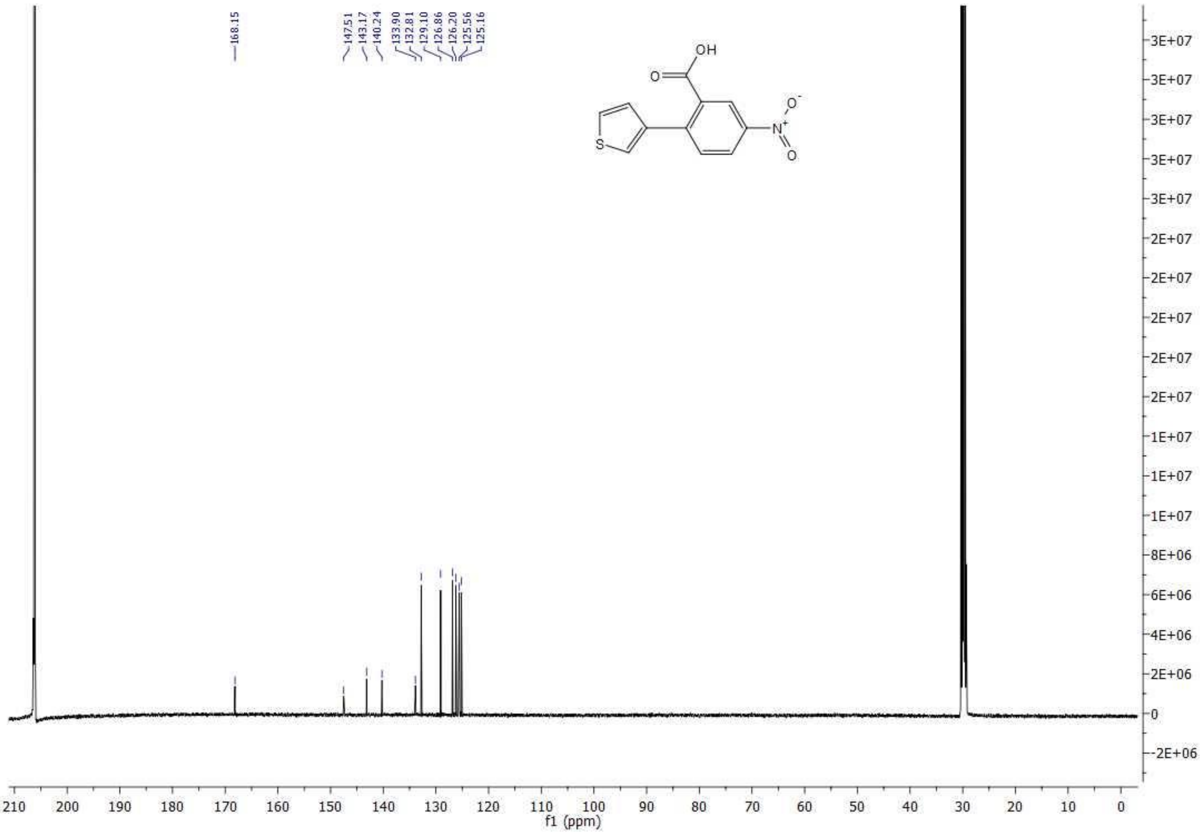
168.15
147.51
143.17
140.24
133.90
132.81
129.10
126.86
126.20
125.56
125.16
OH
O
S
N+
O-
f1 (ppm)

Compound **14**

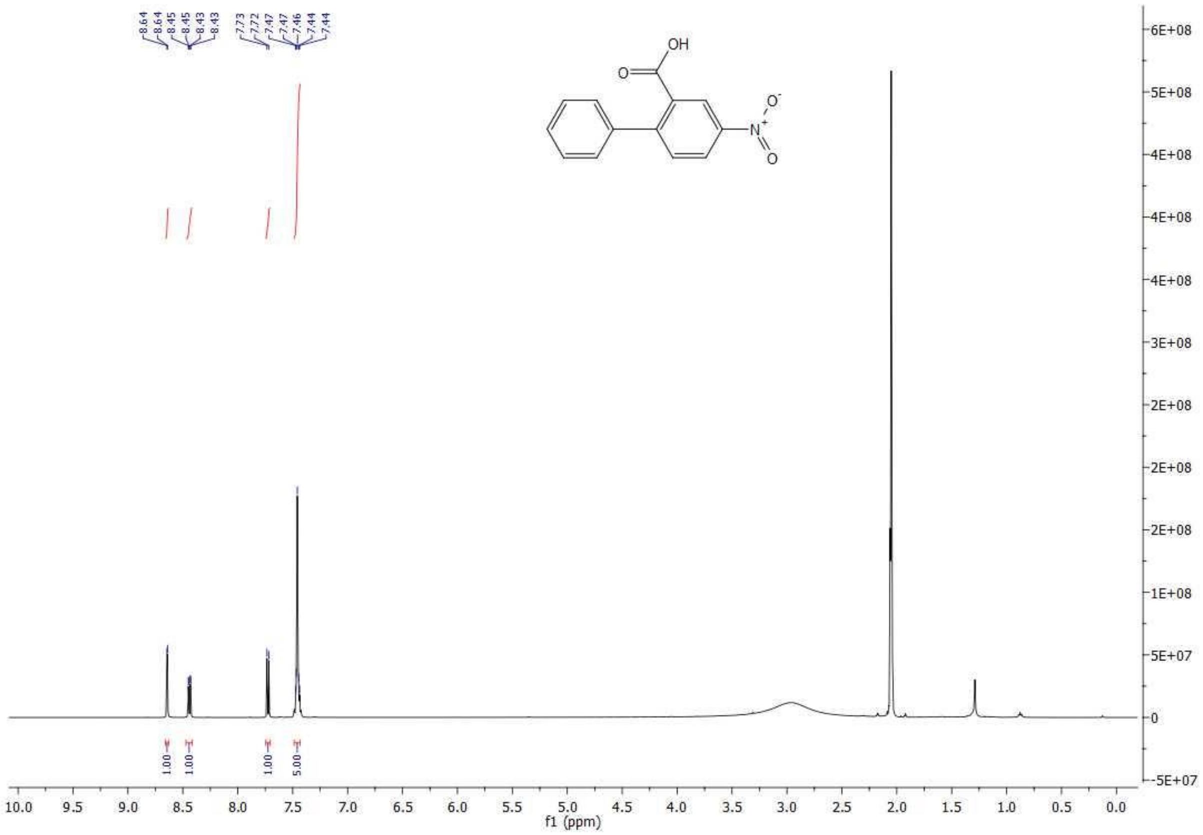

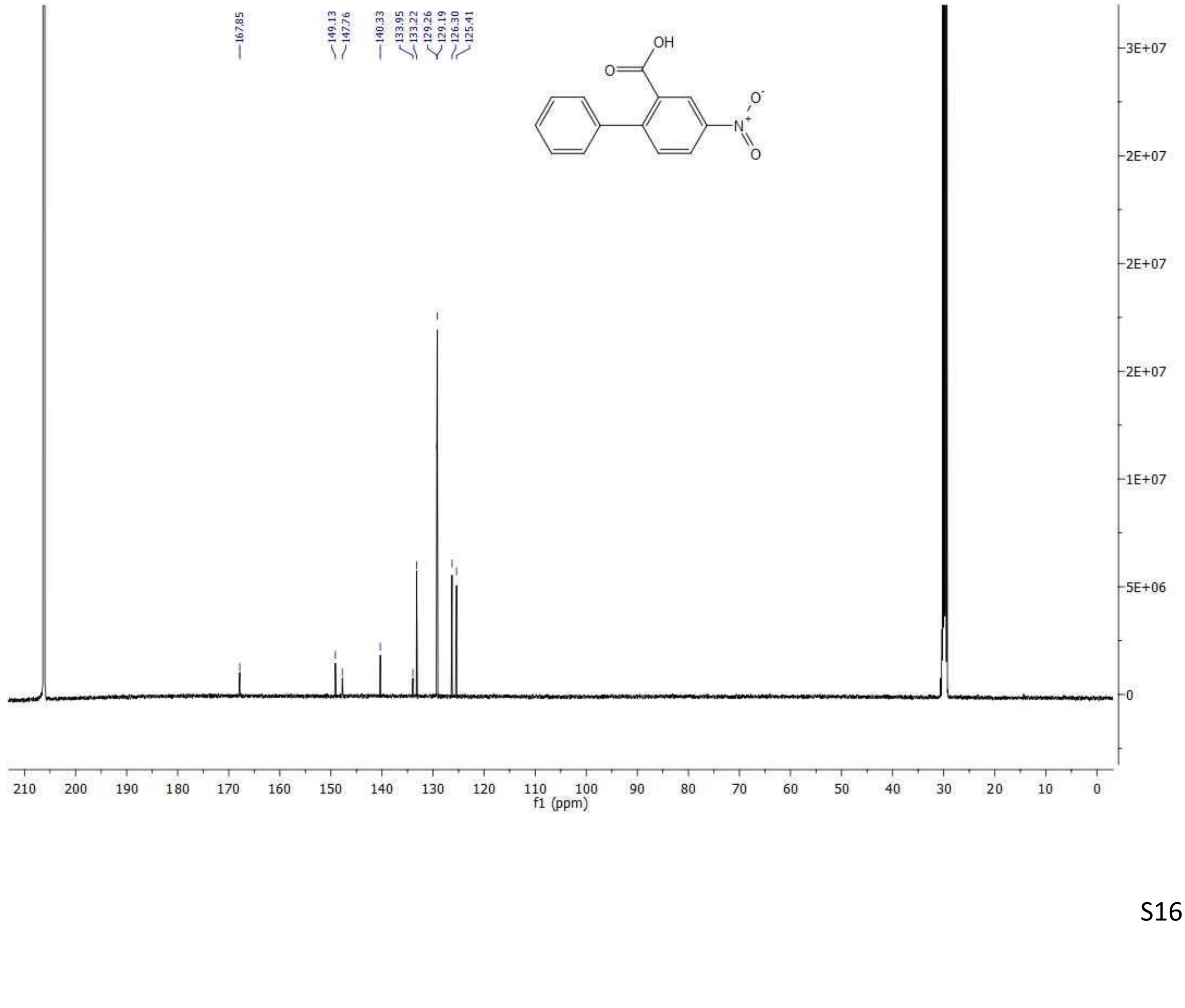

Internal

Compound **15**

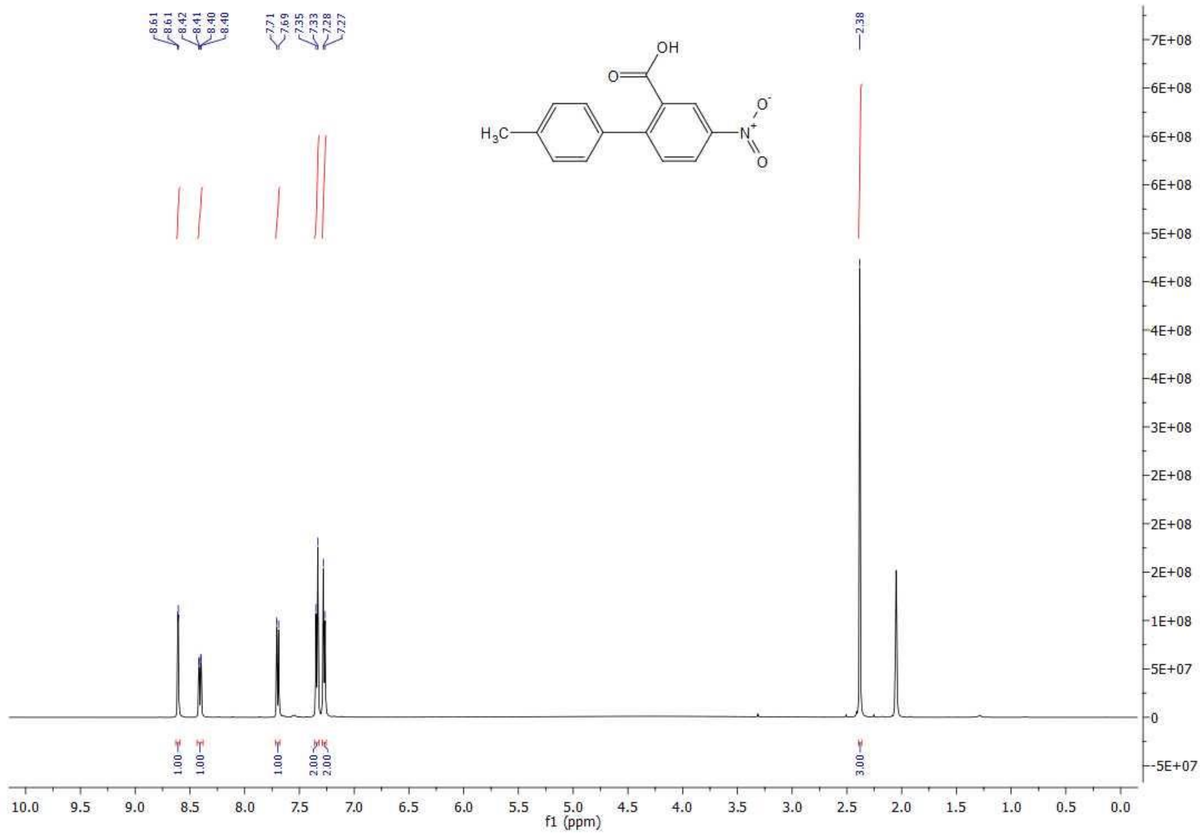

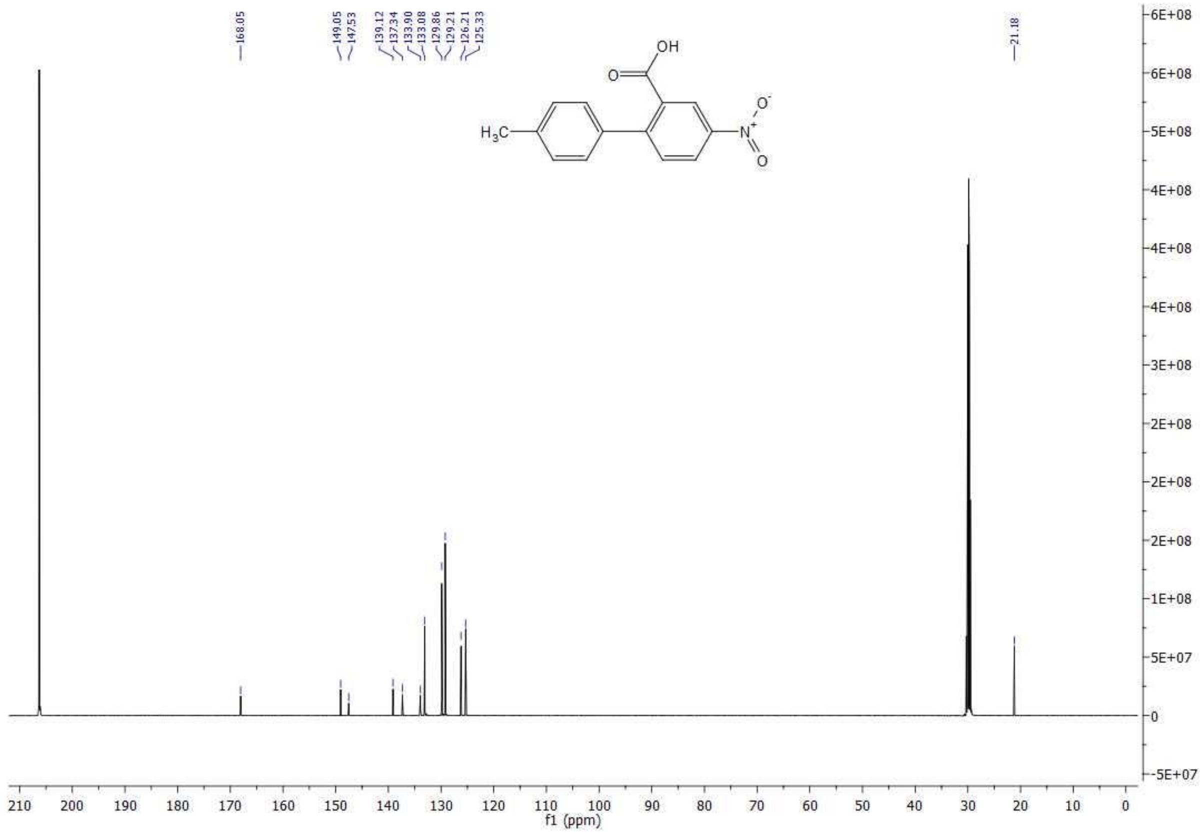

Internal

Compound **16**

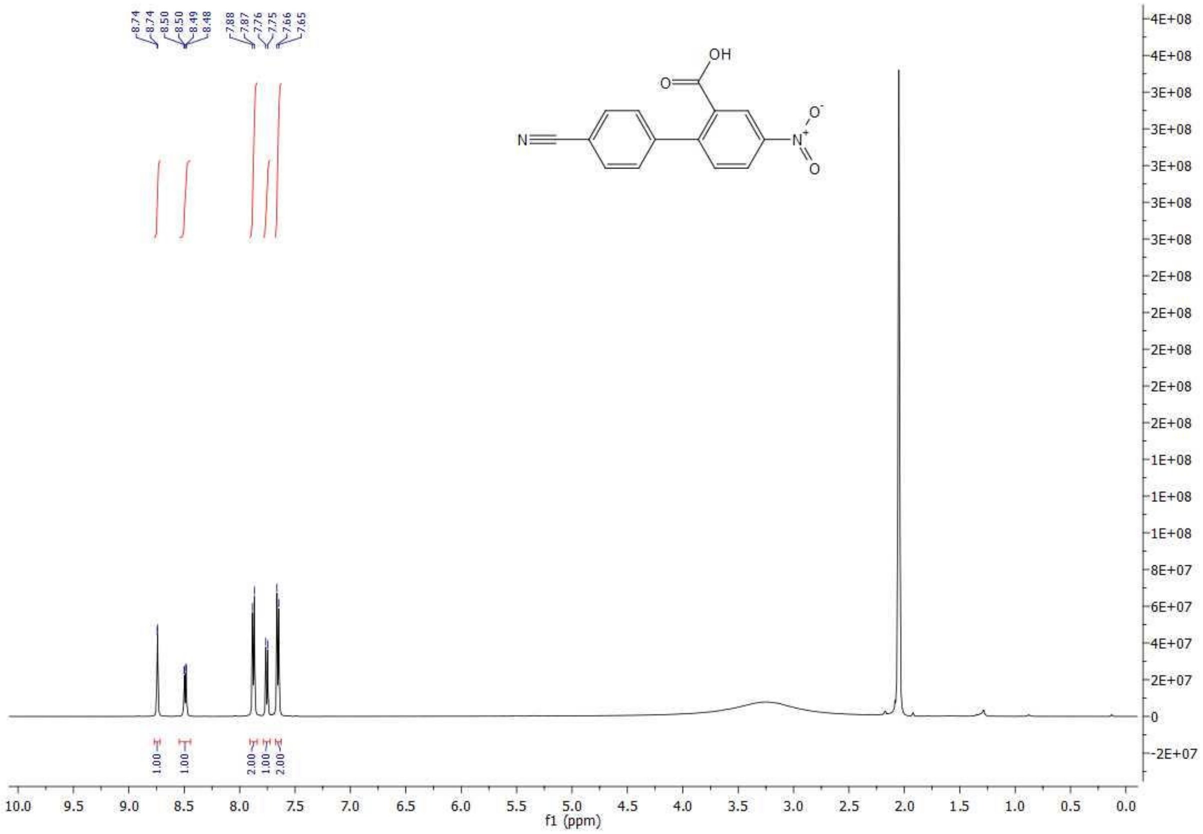

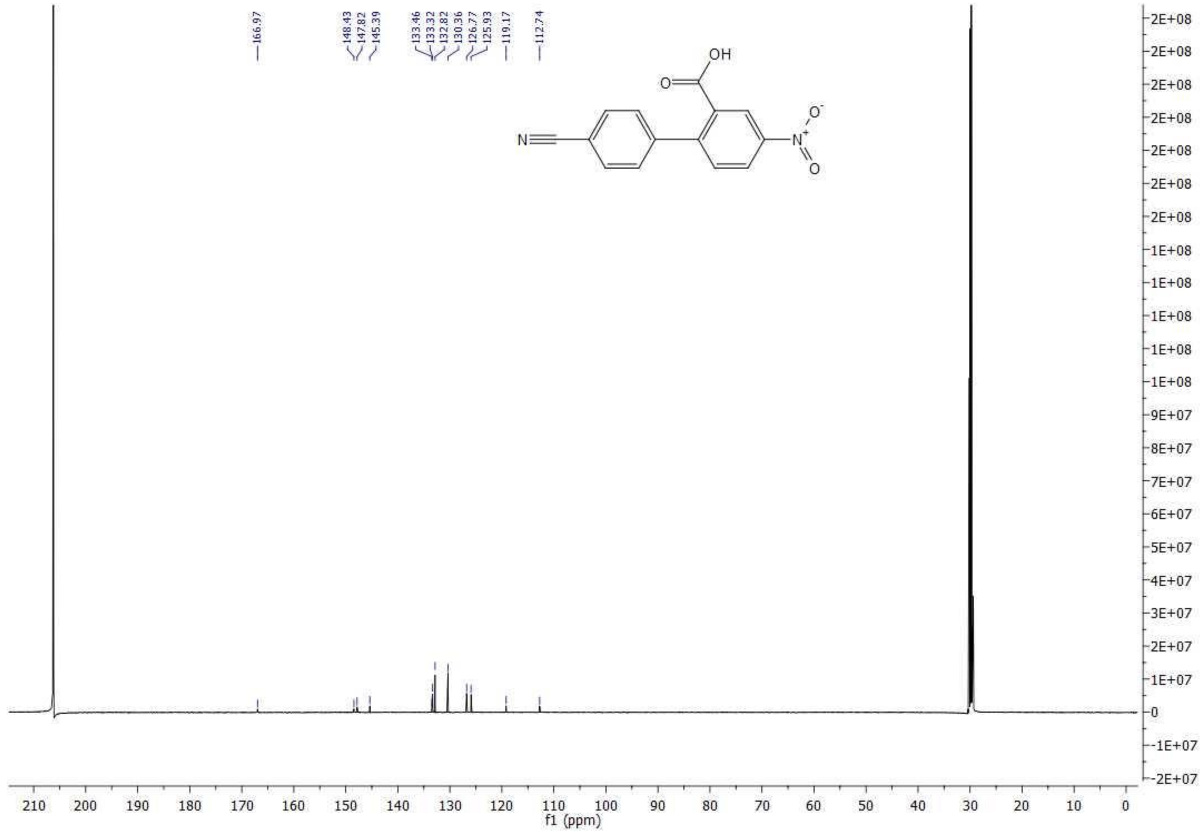

Internal

Compound **17**

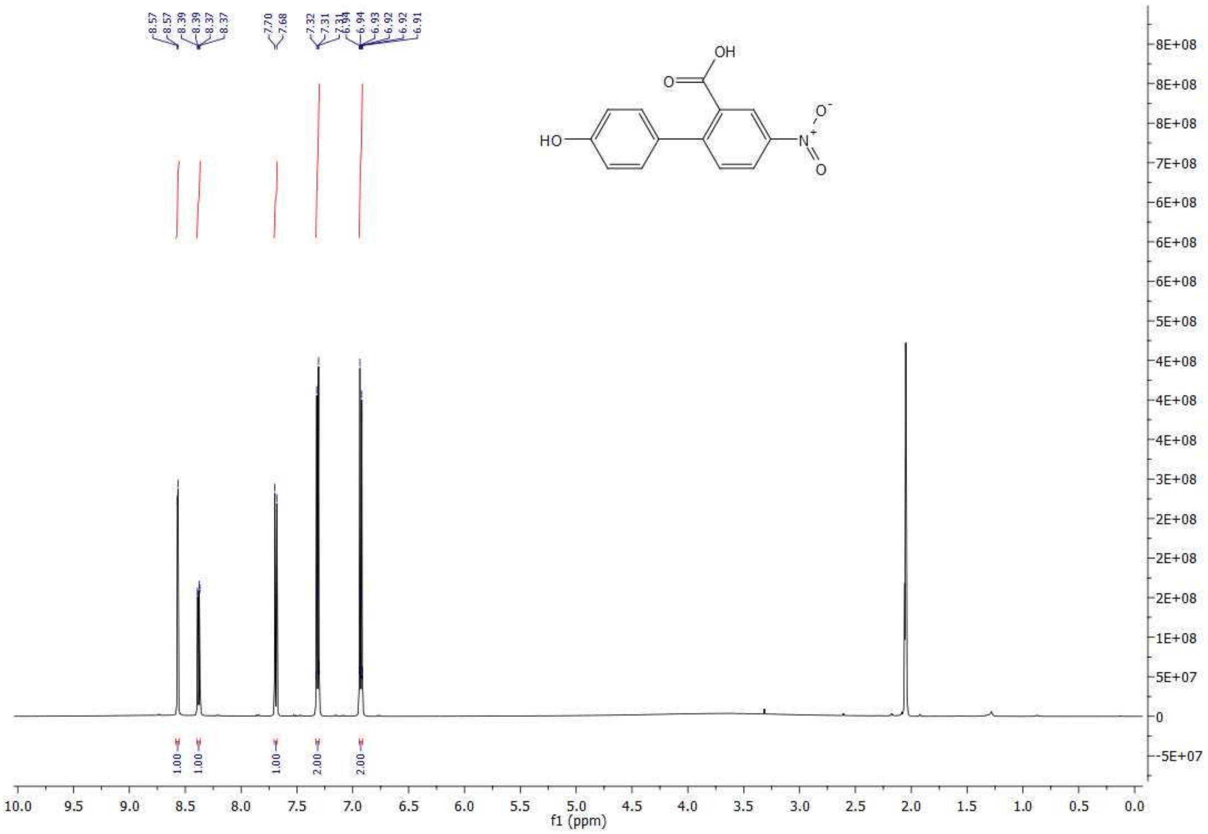

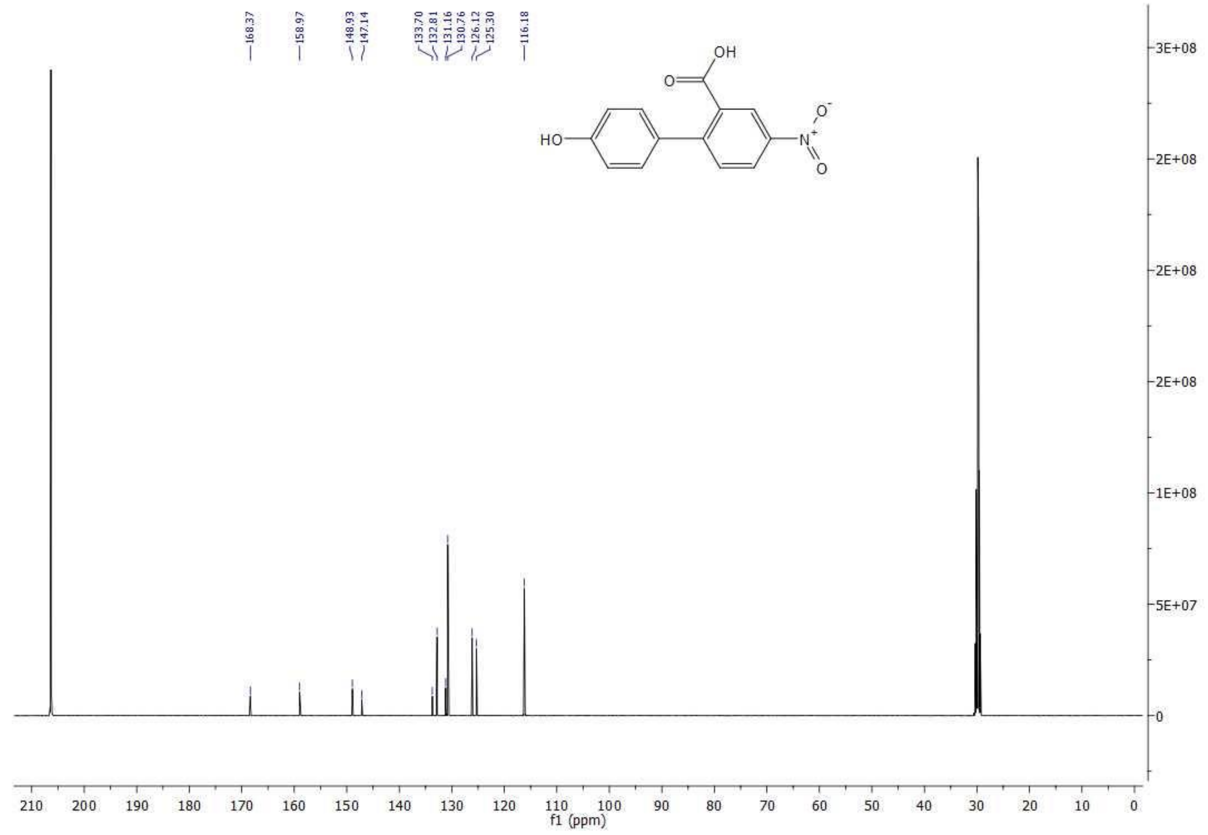

Internal

Compound **18**

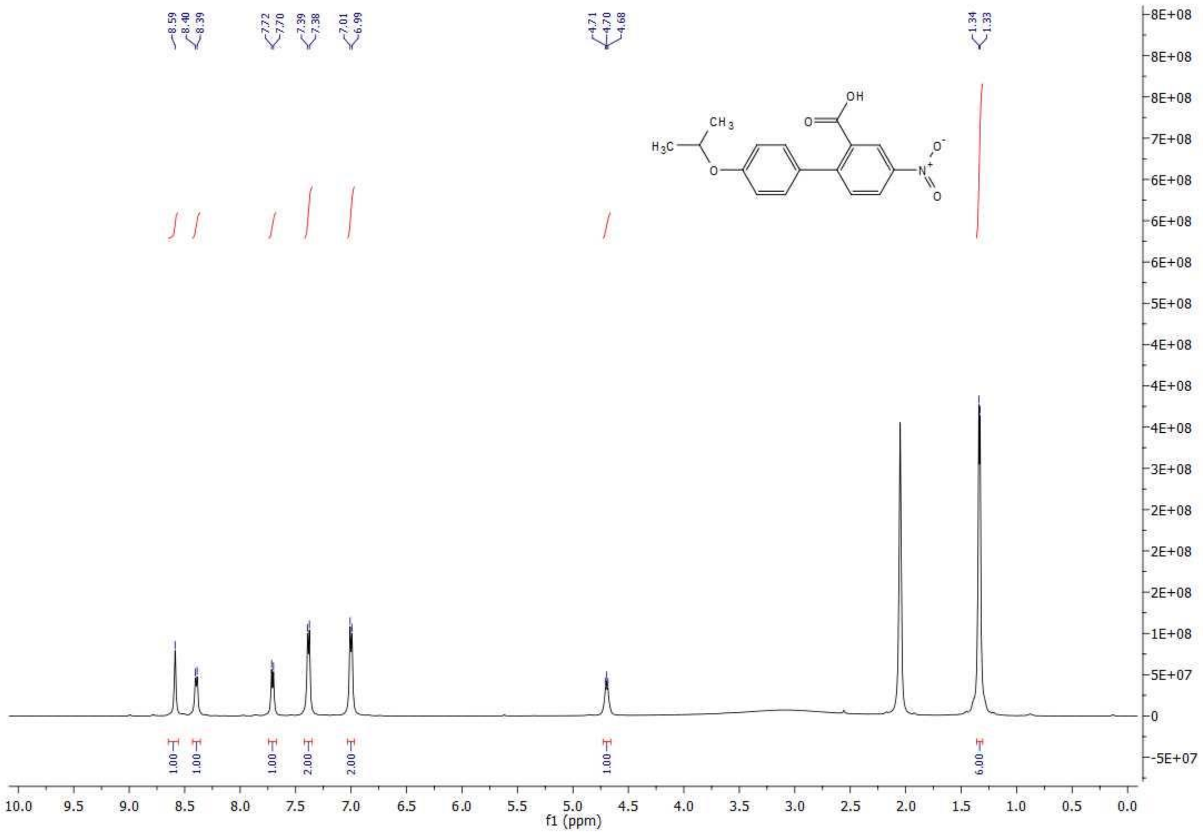

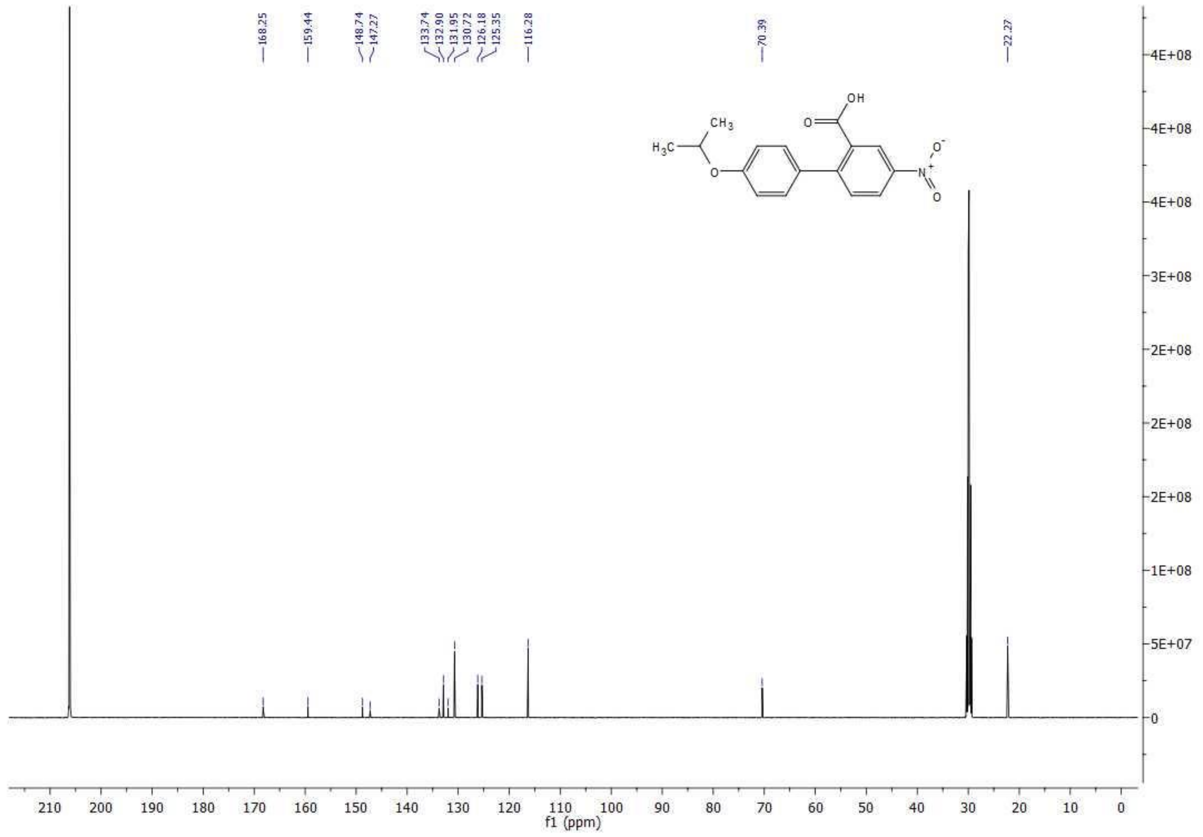

Internal

6 REFERENCES

[1] P. Kuzmic, *Anal. Biochem.* **1996**, *237*, 260–273.

[2] A. Honold, C. Lettl, F. Schindele, B. Illarionov, R. Haas, M. Witschel, A. Bacher, M. Fischer, *Helv. Chim.* **2019**, *102*.

[3] L. A. Kelley, S. Mezulis, C. M. Yates, M. N. Wass, M. J. Sternberg, *Nat. Protoc.* **2015**, *10*, 845–858.

[4] L. S. Imlay, C. M. Armstrong, M. C. Masters, T. Li, K. E. Price, R. L. Edwards, K. M. Mann, L. X. Li, C. L. Stallings, N. G. Berry, P. M. O'Neill, A. R. Odom, *ACS Infect. Dis.* **2015**, *1*, 157–167.

Internal
